# 

**Published:** 1902-12

**Authors:** 


### INDEX TO VOLUME XXIII.

A.

A.B. or M.D., which shall it be? 485
Abscesses, antral, amputation of the palatal roots of the superior first molar for the prevention of, 784
Academy of Stomatology, 245, 508, 590, 668, 763, 833, 927
Actinomycosis, 169
Address, a brief, 909
Adenoids in relation to structural changes, 393
Advertising, burlesque, as a means of exposing fakirs, 617
Air, treatment of pulmonary complaints with, from limestone caves, 865
Allan, Charles F., what should be the relation of our government to the dental profession? 149
  George S., extension for prevention, 311
Alloy, process for producing gold-like, from copper and antimony, 538
Aluminum for dental plates, 801
Alumni Association, New York College of Dentistry, 297
Amalgam, precipitated silver to hasten the setting of, 291
American Academy of Dental Science, 33, 187, 358, 596, 653, 821
  Dental Society of Europe, 457, 543
  Medical Association, Section on Stomatology, 354, 385, 390, 769
  Society of Orthodontists, 300, 778, 858
Amputation of the palatal roots of superior first molars for the prevention of antral abscesses, 784
Anæmia, pernicious, caused by oral sepsis, 699
Anæsthesia, nitrogen monoxide in combination with oxygen for, 387
  local, by high-frequency electrical currents, 698
  of the teeth by means of high-frequency currents, 651
Anæsthetic, a new, somnoform, 747
Anæsthetics, suggestion in the administration of, 293
Anderson, Martha, some notes concerning preparation of teeth for microscopic study, 588
Andrews, R. R., the embryology of the dental pulp, 621
Antimony and copper, process of producing gold-like alloy from, 538
Army, a bill to reorganize the corps of dental surgeons attached to the medical department of, 456
  dentists, the, 221
Arrington, B. F., dentistry as a vocation for profit, especially in North Carolina, 239
  inconsistent and misleading expressions, 490
Arsenical poisoning cured by orthoform, 295
Attachment, concealed bicuspid, for bridge, 538
  concealed gold bridge, 144
Award of the Gross prize of one thousand dollars, 222

**B.**

Babylonians, Chaldeans and, metallurgical skill of, 865
Backings, an original method of adapting to facings, 698
Bacteria on Mont Blanc, 696
  the action of liquid air upon, 696
Baker, L. W., facial and dental harmony, 69
Bands, how to splice engine, 618
Banquet in honor of Dr. S. B. Palmer, of Syracuse, N. Y., 950
Barry, Dr. Burt, in memoriam, 282
Beads, use of, in retaining rubber dam, 866
BIBLIOGRAPHY:
  Abbott's Bacteriology, A Practical Manual for Students and Physicians. By A. C. Abbott, 528
  Anatomy, Descriptive and Surgical. By Henry Gray, 142
  Notes on Materia Medica, Pharmacology, and Therapeutics, for Dental Students and Practitioners. By Douglas Gabell, 530
  Principles and Practice of Operative Dentistry. By John Sayre Marshall, 224
  Questions and Answers. By Ferdinand J. S. Gorgas, 225
  Quiz Compend on Irregularities of the Teeth. By Eugene S. Talbot, 280
  Simon's Manual of Chemistry. By W. Simon, 59
  Studies of the Internal Anatomy of the Face. By M. H. Cryer, 140
  The Röntgen Rays in Medicine and Surgery, as an Aid in Diagnosis and as a Therapeutic Agent. By Francis H. Williams, 276
Billeter's, Professor, report on dental education, 134
Bill to add a corps of dental surgeons to the bureau of medicine and surgery of the navy, 456
  to reorganize the corps of dental surgeons attached to the medical department of the army, 456
Bills before the Senate, Fifty-seventh Congress, 455
BIOGRAPHICAL SKETCHES:
  Dr. Benjamin Lord, 531
  Edward Hudson, 441
  Royal William Varney, 284
Board of Dental Examiners in Pennsylvania, 296, 868
Bogue, E. A., observations on some recent cases of orthodontia, 869
Boston letter, 693, 949
Bradley, Frederick, foreign observations, 15
Bridge, concealed bicuspid attachment for, 538
Briggs, E. C., porcelain inlays, 630
British Dental Association and the National Dental Association of the United States contrasted, 745
Broaches, Swiss, to produce spring temper in, 777
Brown, George V. I., general nervous manifestations in relation to jaws and teeth, 571
  surgical correction of malformation and speech defects due to or associated with harelip or cleft palate, 301

**C.**

Capsule implantations, 372
Carroll, N. G., professional honesty and decision of character in the practice of dentistry, 91
Cataphoresis, the value of, 781
Caves, limestone, treatment of pulmonary complaints with air from, 865

Cavities, to varnish the inside of, without touching the margins with the varnish, 451
Celluloid table-cover, 292
Central Dental Association of Northern New Jersey, 299
Century, the second year of, 945
Chaldeans, metallurgical skill of, and Babylonians, 865
Character, professional honesty and decision of, in the practice of dentistry, 91
Chicago Dental Society, 542
Chittenden, Charles C., the legal status of the term "reptuable" as applied to dental colleges, 581
Chloroform, collapsed while extracting under, 952
Clamps, piano wire, 228
Cleft palate, surgical correction of malformation and speech defects, due to or associated with harelip or, 301
Collapsed while extracting under chloroform, 952
College course and professional training, 156
Colleges, dental, the legal status of the term "reputable" as applied to, 581
Collodion as a model varnish, 454
Colorado State Board of Dental Examiners, 868
    Dental Association, 464, 619
Committees, the two, 859
Congress, Fifty-seventh, bills before the Senate, 455
Connecticut State Dental Association, 296
Conventions, the summer, 436
Copper and antimony process for producing gold-like alloy from, 538
Cork-forests, the great, of Spain, 863
Cork, use of, between the teeth while malleting, 454
Cottonoid strips with the rubber dam, 145
Counter-die and die of Mellotte's metal, 617
Criticism, a: professional education, 217
    putrescent pulps, 861
Crockett, E. A., deaf mutes, 405
Crown, an emergency, 291, 537
    bell-shaped, to be applied without trimming the tooth, 388
    the burnished-cap-, 545
Crown-post, to bend, without strain on the crown, 388
Crowns, gold, carved solid cusp for, 777
CURRENT NEWS:
    Alumni Association, New York College of Dentistry, 297
    American Dental Society of Europe, 457, 543
        Society of Orthodontists, 300, 778
    Bill to add dental surgeons to the navy, 147
    Bills before the Senate, Fifty-seventh Congress, 455
    Board of Dental Examiners in Pennsylvania, 296, 868
    Chicago Dental Society, 542
    Colorado State Board of Dental Examiners, 868
        Dental Association, 464
    Connecticut State Dental Association, 296
    G. V. Black Dental Club, 620
    Harvard Dental Alumni Association, 461, 619
        Odontological Society, 298
    Illinois State Dental Society, 541
    Institute of Dental Pedagogics, 298, 953
    Iowa State Dental Society, 299
    Maine Dental Society, 543
    Mississippi Board of Dental Examiners, 295, 392

Current News:
Missouri State Dental Association, 300, 391, 542
National Association of Dental Examiners, 460, 700
of Dental Faculties, 460, 541
Dental Association, 459, 540
New England Association of Dental Examiners, 460
New Jersey State Board of Dental Examiners, 779
State Board of Registration and Examination in Dentistry, 296
State Dental Society, 146, 458
State Dental Society Meeting, 463
New York Odontological Society, 67
North Carolina State Board of Dental Examiners, 459
Northeastern Dental Association, 779
Northern Illinois Dental Society, 66
Ohio Dental Association, 461
Odontological Society of Chicago, 146
Pennsylvania Association of Dental Surgeons, 68, 954
Board of Dental Examiners, 392
State Dental Society, 461, 541
Reading Dental Society, 297
Seventh District Dental Society of the State of New York, 297
Southern Branch National Dental Association, 67, 148
Tennessee Dental Association, 462, 780

Current News:
Texas State Dental Association, 300, 620
Union Meeting of the Seventh and Eighth District Dental Societies of New York, 780
Virginia State Dental Association, 544
Currents, electrical, local anæsthesia by high-frequency, 698
high-frequency, anæsthesia of the teeth by means of, 651
Curricula, dental college, as related to physical diagnosis, 565
Curtis, G. Lenox, electric ozonation in neuralgia, 635
Cusp, carved solid, for gold crowns, 777

D.

Davenport, S. E., stray thoughts about regulating, 736
Decay, notes on methods of preventing, 888
Degree, a new, needed, 614
Degrees, conferring honorary, 381
De Lisle, Justin, some popular errors about microbes, 469
Dental education, Dr. Guillermin's communication on, 132
surgeons, a bill to add a corps of, to the bureau of medicine and surgery of the navy, 456
a bill to reorganize the corps of, attached to the medical department of the army, 456
Dentine, carious, histological and clinical investigations as to the method in which nitrate of silver affects, 331
obtunding sensitive, by the use of nitrate of silver, 426
the present state of our knowledge in regard to the sensitiveness of, and its treatment, 492

Dentine, the reticulum in, 900
Dentist, the modern, from a medical stand-point, 646
Dentistry, a plea for a sub-specialty in, 235
as a vocation for profit especially in North Carolina, 239
extract of the suprarenal gland in, 905
professional honesty and decision of character in the practice of, 91
Dentists, appointment of, in New York hospitals, 450
Details, importance of, 893
Diagnosing, the use of heat as a means of, the presence of pus, 294
Diagnosis of presence of pus by heat, 699
physical, as related to college curricula, 565
Die and counter-die of Mellotte's metal, 617
Dioxygen, instructions for concentrating, 777
DISCUSSIONS:
A brief address, 911
A comparative study of the attachment of the teeth, 917
A contribution to operative orthodontia, 602
A peculiar case of dental resorption, 763
Adenoids in relation to structural changes, 421
Among the Honduranians, ancient and modern, 360
Amputation of the palatal roots of superior first molars for the prevention of antral abscesses, 821
Capsule implantation, 373
Deaf mutes, 429
Dental education, 205
Electric ozonation in neuralgia, 921
Evolution of the pulp, 846
DISCUSSIONS:
Extension for prevention, 345
Facial and dental harmony, 114
Faulty environment, 33
Good of the order, 370
Importance of details, 908
Incidents of office practice, 668
Kirk's, E. C., remarks on Dr. Michael's work, 246
Notes on methods of preventing decay, 913
Observations on some recent cases of orthodontia, 918
Oral hygiene, 925
Physical diagnosis as related to dental college curricula, 677
Physiological results of operation for cleft palate, 600
Porcelain inlays, 659
President's address, 941
Preventive dentistry, 175
Résumé of the histology of the dental pulp, 840
Science as a teacher of prophylaxis, 104
Some notes concerning preparation of teeth for microscopic study, 769
Some peculiar cases of dental resorption, 508
Some popular errors about microbes, 502
Some things I have found useful in the practice of dentistry, 516
Stomatitis from a dental standpoint, 927
Stray thoughts about regulating, 754
Surgical correction of malformation and speech defects due to or associated with harelip and cleft palate, 354
The burnished-cap-crown, 590
The college course and professional training, 190
The control of our patients, 41

DISCUSSIONS:
- The hospitals need of a dental staff, 803
- The modern dentist from a medical stand-point, 922
- The value of cataphoresis, 833
- To restore the cutting or grinding surfaces of abraded teeth with gold, 501
- What should be the relation of our government to the dental profession, 172

Disease, flies as carriers of, 864

Disinfection of dental instruments by spirits of soap, 169

DOMESTIC CORRESPONDENCE:
- Appointments of dentists in New York hospitals, 450
- Banquet in honor of Dr. S. B. Palmer, of Syracuse, N. Y., 950
- Boston letter, 693, 949
- Incident of office practice, 143
- Letter from New York City, 116, 287, 386, 447
- Nitrogen monoxide in combination with oxygen for anæsthesia, 387
- Piano wire clamps, 228
- Putrescent pulps: a criticism, 861
- Some popular errors about microbes, 386
- Who first filled pulp-canals? 536

E.

Eames, George F., oral hygiene, 881

EDITORIAL:
- A new degree needed, 614
- A sub-specialty for women, 272
- American Medical Association, Section on Stomatology, 385
  - Society of Orthodontists, 858
- Award of the Gross prize of one thousand dollars, 222

EDITORIAL:
- Biographical sketches, 438
- Conferring honorary degrees, 381
- Death of Dr. H. B. Noble, 276
- Dr. A. W. Harlan retires, 223
- Dr. Benjamin Lord, 440
- Dr. R. W. Morgan retired, 59
- Explanation and apology, 949
- From trade ideas to professional ideals, 135
- International Dental Congress, 946
- Is spelling an important factor in examination? 56
- Newspaper enterprise, 948
- Organizations, present and future, 773
- Professional education: a criticism, 217
  - recreation, 611
- Professor Willoughby D. Miller, 528
- The army dentist, 221
- The Massachusetts Dental Society, annual meeting, 525
- The multiplication of dental associations, 854
- The National Association of Dental Faculties, 691
  - Dental Association, 689
- The Regents of New York and the D.D.S. degree, 521
- The second year of the century, 945
- The summer convention, 436
- The two committees, 859
- The vicissitudes of dental journalism, 853
- What is the true standard of mental training? 54
- Where an explanation seems necessary, 136

Education, dental, discussions on, 205
- Professor Billeter's report on, 134

Education, dental, report by Professor Mêtral on, 135
report on, 125, 206
professional: a criticism, 217
Eighth District Dental Societies of New York, Union Meeting of the Seventh and, 780
Embryology, the, of the dental pulp, 621
Engs, John S., the reticulum in dentine, 900
Enterprise, newspaper, 948
Environment, faulty, 1
Erosion, the use of a compound of milk of magnesia and precipitated chalk for, 426
Errors, some popular, about microbes, 469
Essig, Charles J., obituary, 61
Eucalypto-percha, a method of making, 618
Evolution of the pulp, Eugene S. Talbot, 846
Examination, is spelling an important factor in an? 56
Explanation, where an, seems necessary, 136
Expressions, inconsistent and misleading, 490
Extension for prevention, 311

F.

Facings, an original method of adapting backings to, 698
Fakirs, exposing, burlesque advertising as a means of, 617
Fillebrown, Thomas, physiological results of operation for cleft palate, 557
Fillings, making approximal amalgam, 332
Flanagan, A. J., the hospital's need of a dental staff, 796
true professional life a fine art, 250
Flies as carriers of disease, 864
"Fox and Harris," a few extracts from, in contrast, 21
Fuller, D. A., a brief address

G.

Genese, D., stomatitis from a dental stand-point, 644
Gerrish, Chas. H., good of the order, 367
G. V. Black Dental Club, 620
Ginger as a remedy, 295
Gold fillings in the Mosaic period, 453
Good of the order, 367
Gordon, George Byron, among the Honduranians, 321
Grant, Harry L., which shall it be, M.D., or A.B.? 485
Gross prize of one thousand dollars, award of the, 222
Guillermin's communication on dental education, 132

H.

Hands, to remove the odor of iodoform from, 863
Harelip, surgical correction of malformation and speech defects due to or associated with, or cleft palate, 301
Harlan, A. W., retires, 223
Harmony, facial and dental, 69
Harvard Dental Alumni Association, 40, 461, 619
Odontological Society, 114, 278, 429, 515
Health, general, the dental profession in relation to, 402
Heat, diagnosis of presence of pus by, 699
the use of, as a means of diagnosing the presence of pus, 294
Helps to success in practice, 11
Histology, résumé of the, of the dental pulp, 719

Honduranians, ancient and modern, among the, 321
Honesty, professional, and decision of character in the practice of dentistry, 91
Hopkins, Samuel A., science as a teacher of prophylaxis, 80
Hospitals, general, a note on the appointment of dental surgeons to, 794
    New York, appointment of dentists in, 450
Howard, W. R., an unusual operation, 9
Howe, J. Morgan, notes on methods of preventing decay, 888
Hudson, Edward, biographical sketch, 441
Hydrogen dioxide for powder-stains, 451
Hygiene, oral, 881
Hypodermic needle, to prevent pain when inserting the, 293

I.

Ideals, professional, from trade ideas to, 135
Ideas, from trade, to professional ideals, 135
Illinois State Dental Society, 541
Impression material, a new, 454
Inglis, Charles S., resolutions of respect to, 227
    Otto E., some peculiar cases of dental resorption, 479
Inlays, an improved method of making the matrix for irregular-shaped porcelain, 419
    gold, 867
    porcelain, 630
    vulcanite, 867
Inoculations, antityphoid, results obtained by, 867
Institute of Dental Pedagogics, 298, 953
Instruments, dental, disinfection of, by spirits of soap, 169
International Commission of Education, 205
    Dental Congress, 946
        Federation, 123, 205
            and International Commission of Education, 848
            meetings of Executive Council, Subcommittee, and International Commission of Education, 43
Iodoform, to remove the odor of, from the hands, 863
Iowa State Dental Society, 299

J.

Jack, Louis, a peculiar case of resorption, 633
    the value of cataphoresis, 781
Jackson, Harvey N., the value of the Wenker rubber jaw in teaching therapeutics, 562
Japanese grass-line, separation of teeth with, 39
Jaws and teeth, general nervous manifestations in relation to, 571
Jenkins, N. S., the future of porcelain work, 903
Journalism, dental, vicissitudes of, 853

K.

Kelley, Henry A., the control of our patients, 26
Kirk, Edward C., some account of the work of Dr. Michaels, 229
Knight, William, the modern dentist from a medical stand-point, 646

L.

Latham, V. A., résumé of the histology of the dental pulp, 719
Lett, Isidore, amputation of the palatal roots of superior first molars for the prevention of antral abscesses, 784

Lewis, F. Park, adenoids in relation to structural changes, 393
Life, true professional, a fine art, 250
Linton, Charles C., ventilation of a dental office, 413
Liquid air, the action of, upon bacteria, 696
Lord, Benjamin, 440
biographical sketch, 531
Lubricant, an oil-stone, 777

M.

McCullough, P. B., the burnished-cap-crown, 545
Macleod, Alexander, the dental profession in relation to the general health, 402
Macvane, S. M., the college course and professional training, 156
Maine Dental Society, 543
Malformation, surgical correction of, and speech defects due to or associated with harelip or cleft palate, 301
Manifestations, general nervous, in relation to the jaws and teeth, 571
Massachusetts Dental Society, 250, 367, 932
annual meeting, 525
Matrix, an improved method of making the, for irregular-shaped porcelain inlays, 419
fastening a, with oxyphosphate, 453
M.D. or A.B., which shall it be? 485
Mellotte's metal, die and counter-die of, 617
Membrana tympani, facial neuralgia due to a hair irritating the, 292
Memoriam, in,—Z. T. Sailer, 66
Metallurgical skill of Chaldeans and Babylonians, 865
Métral's, Professor, report on dental education, 135
Michaels, Dr., some account of the work of, 229
work, E. C. Kirk's remarks on, 246
Microbes, some popular errors about 386, 469
Miller, Willoughby D., 528
MISCELLANY:
A method of making eucalypto-percha, 618
A new impression material, 454
method of closing superficial incised wounds, 389
An emergency crown, 291, 537
An oil-stone lubricant, 777
An original method of adapting backings to facings, 698
Arsenical poisoning cured by orthoform, 295, 617
Bacteria on Mont Blanc, 696
Bell-shaped crown to be applied without trimming the tooth, 388
Burlesque advertising as a means of exposing fakirs, 617
Care of the teeth, 451
Carved solid cusp for gold crowns, 777
Celluloid table-cover, 292
Collapsed while extracting under chloroform, 952
Collodion as a model varnish, 454
Concealed gold bridge attachment, 144
bicuspid attachment for bridge, 538
Cottonoid strips with the rubber dam, 145
Diagnosis of presence of pus by heat, 699
Die and counter-die of Mellotte's metal, 617
Facial neuralgia due to a hair irritating the membrana tympani, 292
Fastening a matrix with oxyphosphate, 453

MISCELLANY:
Flies as carriers of disease, 864
Ginger as a remedy, 295
Gold fillings in the Mosaic period, 453
inlays, 867
Holding patients under the influence of nitrous oxide gas, 866
How to splice engine bands, 618
Hydrogen dioxide for powder stains, 451
Instructions for concentrating dioxygen, 777
Local anæsthesia by high-frequency electrical currents, 698
Metallurgical skill of Chaldeans and Babylonians, 865
Parr's fluxed wax, 295
Percentage solutions, 539
Pernicious anæmia caused by oral sepsis, 699
Precipitated silver to hasten setting of amalgam, 291
Process for producing gold-like alloy from copper and antimony, 538
Results obtained by antityphoid inoculations, 867
Soapstone, 145
Suggestion in the administration of anæsthetics, 293
Synthol, 293
Test for adulterated zinc salts, 455
The action of liquid air upon bacteria, 697
The great cork-forests of Spain 863
The use of heat as a means of diagnosing the presence of pus, 294
To bend a crown-post without strain on the crown, 388
To prevent pain when inserting the hypodermic needle, 293

MISCELLANY:
To prevent unsoldering, 145, 293
To produce spring temper in Swiss broaches, 777
To remove the odor of iodoform from the hands, 863
vulcanite from between teeth, 867
To varnish the inside of cavities without touching the margins with the varnish, 451
Treatment of pulmonary complaints with air from limestone caves, 865
Use of beads in retaining rubber dam, 866
of cork between the teeth while malleting, 454
of preservatives for solutions of suprarenal extract, 452
Vulcanite inlays, 867
Mississippi Board of Dental Examiners, 295, 392
Missouri State Dental Association, 300, 391, 542
Models, the making of, which are durable, and which can be painted, 31
Molars, superior first, amputation of the palatal roots of, for the prevention of antral abscesses, 784
Mont Blanc, bacteria on, 696
Morgan, R. W., retired, 59
W. H., resolutions of respect to—Tennessee Dental Association, 65
Mutes, deaf, 405

N.

National Association of Dental Examiners, 460, 700
Faculties, 460, 541, 691

National Dental Association, 540
of the United States, and the British Dental Association contrasted, 745
Southern Branch, 148
Navy, a bill to add a corps of dental surgeons to the bureau of medicine and surgery of, 456
bill to add dental surgeons to the, 147
Necrosis, a case of arsenical, 790
Neuralgia, electric ozonation in, 635
facial, due to a hair irritating the membrana tympani, 292
New England Association of Dental Examiners, 460
New Jersey State Board of Dental Examiners, 779
State Board of Registration and Examination in Dentistry, 296
State Dental Society, 146, 458
State Dental Society, Meeting of, 463
Newkirk, Garrett, review of E. S. Talbot's paper, "A study of the degeneracy of the jaws of the human race," 332
New York Institute of Stomatology, The, 99, 172, 332, 421, 501, 749, 802, 907
Odontological Society, 67
Nitrate of silver and eucaine for obtunding sensitive dentine, 426
histological and clinical investigations as to the method in which, affects carious dentine, 331
Nitrogen monoxide in combination with oxygen for anæsthesia, 387
Nitrous oxide gas, holding patients under the influence of, 866
Noble, Henry Bliss, death of, 276
obituary of, 282
North Carolina State Board of Dental Examiners, 459
Northeastern Dental Association, 779
Northern Illinois Dental Society, 66
Ohio Dental Association, 461
Notes, dental, 893
Noyes, Frederick B., a comparative study of the attachment of the teeth, 639

O.

Obituary:
Essig, Charles J., M.D., D.D.S., 61
In memoriam, Dr. Burt Barry, 282
Noble, Henry Bliss, 282
Resolutions of respect to Dr. Charles S. Inglis, 227
Tennessee Dental Association—resolutions of respect to Dr. W. H. Morgan, 65
Wetherbee, Dr. Isaac J., 695
Observations, foreign, 15
on some recent cases of orthodontia, 869
Odontological Society of Chicago, 146
Operation, an unusual, 91
Organizations, present and future, 773
Original Communications:
A case of arsenical necrosis, 790
A comparative study of the attachment of the teeth, 639
A contribution to operative orthodontia, 553
A few extracts from "Fox and Harris" in contrast, 21
A note on the appointment of dental surgeons to general hospitals, 794
A peculiar case of resorption, 633
A plea for a sub-specialty in dentistry, 235

ORIGINAL COMMUNICATIONS:
Adenoids in relation to structural changes, 393
Among the Honduranians, ancient and modern, 321
Amputation of the palatal roots of superior first molars for the prevention of antral abscesses, 784
An unusual operation, 9
Deaf mutes, 405
Dental notes, 893
Dentistry as a vocation for profit, especially in North Carolina, 239
Electric ozonation in neuralgia, 635
Evolution of the pulp, 701
Extension for prevention, 311
Facial and dental harmony, 69
Faulty environment, 1
Foreign observations, 15
General nervous manifestations in relation to the jaws and teeth, 571
Helps to success in practice, 11
Importance of details, 893
Inconsistent and misleading expressions, 490
Notes on methods of preventing decay, 888
Observations on some recent cases of orthodontia, 869
Oral hygiene, 881
Physical diagnosis as related to dental college curricula, 565
Physiological results of operation for cleft palate, 557
Porcelain inlays, 630
Professional honesty and decision of character in the practice of dentistry, 91
Résumé of the histology of the dental pulp, 719
Science as a teacher of prophylaxis, 80
Some account of the work of Dr. Michaels, 229

ORIGINAL COMMUNICATIONS:
Some notes concerning preparation of teeth for microscopic study, 588
Some peculiar cases of dental resorption, 479
Some popular errors about microbes, 469
Some things I have found useful in practice, 465
Stomatitis from a dental standpoint, 644
Stray thoughts about regulating, 736
Surgical correction of malformation and speech defects due to, or associated with, harelip or cleft palate, 301
The British Dental Association and the National Dental Association of the United States contrasted, 745
The burnished-cap-crown, 545
The college course and professional training, 156
The coming physiology: a prediction, 897
The control of our patients, 26
The dental profession in relation to the general health, 402
The embryology of the dental pulp, 621
The future of porcelain work, 903
The hospital's need of a dental staff, 796
The legal status of the term "reputable" as applied to tal colleges, 581
The modern dentist from a medical stand-point, 646
The reticulum in dentine, 900
The value of cataphoresis, 781
of the Wenker rubber jaw as an aid in teaching therapeutics, 562

Original Communications:
To restore the cutting or grinding surfaces of abraded teeth with gold, 483
Ventilation of a dental office, 413
What should be the relation of our government to the dental profession? 149
Which shall it be, M.D. or A.B.? 485
Orthodontia, operative, a contribution to, 553
observations on some recent cases of, 869
Orthoform, arsenical poisoning cured by, 295, 617
Oxyphosphate, fastening a matrix with, 453
Ozonation, electric, in neuralgia, 635

P.

Pain, to prevent, when inserting the hypodermic needle, 293
Palate, cleft, physiological results of operation for, 557
Palmer, S. B., the coming physiology: a prediction, 897
banquet in honor of, 950
Parmele, Geo. L., helps to success in practice, 11
Patients, holding, under the influence of nitrous oxide gas, 866
the control of our, 26
Peck, A. H., physical diagnosis as related to dental college curricula, 565
Pennsylvania Association of Dental Surgeons, 68, 954
Board of Dental Examiners, 391
State Dental Society, 461, 541
Physiology, the coming: a prediction, 897
Piano wire clamps, 228
Pin of Logan crown crystallized, 358
Plates, dental, aluminum for, 801
Poisoning, arsenical, cured by orthoform, 617
Porcelain, the practical value of, as a filling material, 243
work, the future of, 903
Port, G., the making of models which are durable, and which can be painted, 31
Powder-stains, hydrogen dioxide for, 451
Power, James Edward, a case of arsenical necrosis, 790
Practice, helps to success in, 11
office, incident of, 143
some things I have found useful in, 465
Prediction, a: the coming physiology, 847
Prevention, extension for, 311
Profession, dental, what should be the relation of our government to the? 149
the dental, in relation to the general health, 402
Professional education: a criticism, 217
training, the college course and, 156
Prophylaxis, science as a teacher of, 80
Pulmonary complaints, treatment of, with air from lime-stone caves, 865
Pulp-canals, who first filled? 536
Pulp, dental, résumé of the histology of the, 719
the embryology of the, 621
evolutions of, 701
Pulps, putrescent: a criticism, 861
Pus, diagnosis of presence of, by heat, 699
the use of heat as a means of diagnosing the presence of, 294
Pyorrhœa alveolaris, progress report, 494

R.

Reading Dental Society, 277

Regents, the, of New York and the D.D.S. degree, 521

Regulating, stray thoughts about, 736

Remedy, ginger as, 295

REPORTS OF SOCIETY MEETINGS:

Academy of Stomatology, 245, 508, 590, 668, 763, 833, 927

Address, annual, New Jersey State Dental Society, 685

American Academy of Dental Science, 33, 187, 358, 596, 653, 821

Medical Association, Section on Stomatology, 354, 608, 677, 763, 840

Harvard Dental Alumni Association, 40

Odontological Society, 114, 429, 515

International Commission of Education, 205

Dental Federation, 123, 205

Dental Federation and International Commission of Education, 848

Dental Federation: Meetings of Executive Council, Subcommittee, and International Commission of Education, 43

Massachusetts Dental Society, 250, 367, 932

New Jersey State Dental Society, 684

Practice, incidents of, 513, 653

The New York Institute of Stomatology, 99, 172, 332, 421, 501, 749, 802, 907

Theory and practice, communications on, 100, 332, 426, 750, 907

"Reputable," the legal status of the term, as applied to dental colleges, 581

Resolutions of respect to Dr. W. H. Morgan—Tennessee Dental Association, 65

Resorption, a peculiar case of, 633

dental, some peculiar cases of, 479

Results, physiological, of operation for cleft palate, 557

Reticulum, the, in dentine, 900

Review, a short, of E. S. Talbot's paper, "A study of the degeneracy of jaws of the human race," 332

REVIEWS OF DENTAL LITERATURE:

Actinomycosis, 169

Aluminum for dental plates, 80

Anæsthesia of the teeth by means of high-frequency currents, 651

An improved method of making the matrix for irregular-shaped porcelain inlays, 419

Extract of the suprarenal gland in dentistry, 905

Histological and clinical investigation as to the method in which nitrate of silver affects carious dentine, 331

Pyorrhœa alveolaris, progress report, 494

Somnoform, a new anæsthetic, 747

The disinfection of dental instruments by spirits of soap, 169

The making of models which are durable, and which can be painted, 31

The practical value of porcelain as a filling-material, 243

The present state of our knowledge in regard to the sensativeness of dentine and its treatment, 492

The use of suprarenal in dental cases, 413

Rollins, William, dental notes, 893

Roots, amputation of the palatal, of superior first molars for prevention of antral abscesses, 784
Rubber dam, cottonoid strips with, 145
 use of beads in retaining, 866

S.

Sailer, Z. T., in memoriam, 66
Science as a teacher of prophylaxis, 80
Sepsis, oral, pernicious anæmia caused by, 699
Seventh and Eighth District Dental Societies of New York, Union Meeting of, 780
 District Dental Society of the State of New York, 297
Silver, precipitated, to hasten the setting of amalgam, 291
Sketches, biographical, 438
Smith, Eugene H., a contribution to operative orthodontia, 553
 F. Milton, to restore the cutting or grinding surfaces of abraded teeth with gold, 483
Soap, spirits of, disinfection of dental instruments by, 169
Soapstone, 145
Solutions, percentage, 539
Somnoform, a new anæsthetic, 747
Southern Branch National Dental Association, 67
Speech defects, surgical correction of malformation and, due to or associated with harelip and cleft palate, 301
Spelling, is, an important factor in examination? 56
Spencer, H. C., a few extracts from "Fox and Harris" in contrast, 21
Staff, dental, the hospital's need of a, 796
Status, the legal, of the term "reputable," as applied to dental colleges, 581
Stoddard, A. H., some things I have found useful in practice, 465
Stomatitis from a dental standpoint, 644
Study, a comparative, of the attachment of the teeth, 635
Sub-specialty for women, a, 272
 in dentistry, a plea for a, 235
Suprarenal extract, use of preservatives for solutions of, 452
 in dentistry, 905
 the use of, in dental cases, 413
Surgeons, dental, a note on the appointment of, to general hospitals, 794
 bill to add, to the navy, 147
Synthol, 293

T.

Table-cover, celluloid, 292
Talbot, Eugene S., evolutions of the pulp, 701
Teeth, abraded, to restore the cutting or grinding surfaces of, with gold, 483
 a comparative study of the attachment of, 639
 anæsthesia of, by means of high-frequency currents, 651
 and jaws, general nervous manifestations in relation to, 571
 the care of the, 451
 separation of, with Japanese grass-line, 39
 some notes concerning preparation of, for microscopic study, 588
 to remove vulcanite from between, 867
 use of cork between, while malleting, 454
Temper, to produce spring, in Swiss broaches, 777
Tennessee Dental Association, 462, 780

Tennessee Dental Association—resolutions of respect to Dr. W. H. Morgan, 65
Test for adulterated zinc salts, 453
Texas State Dental Association, 300, 620
The hospital's need of a dental staff, 796
The New York Institute of Stomatology, 99, 172, 332, 421, 501, 749, 802, 907
Theory and practice, communications on, 100, 332, 426, 750, 907
Therapeutics, the value of the Wenker rubber jaw as an aid in teaching, 562
Training, mental, what is the true standard of? 54
Trueman, William H., the British Dental Association and the National Dental Association of the United States contrasted, 745

U.

Union Meeting of the Seventh and Eighth District Dental Societies of New York, 780
Unsoldering, to prevent, 145, 293

V.

Varney, Royal William, 284
Varnish, collodion as a model, 454
Ventilation of a dental office, 413
Virginia State Dental Association, 544
Vulcanite, to remove, from between teeth, 867

W.

Wax, Parr's fluxed, 295
Wedelstaedt, E. K., faulty environment, 1
Weiser and Zsigmondy's report on dental education, 206
Wenker rubber jaw, the value of, as an aid in teaching therapeutics, 562
Wetherbee, Isaac J., obituary, 695
Whitlock, W. M., importance of details, 893
Williams, E. Lloyd-, a note on the appointment of general surgeons to general hospitals, 794
Women, a sub-specialty for, 272
Wounds, a new method of closing superficial incised, 389
Wright, C. M., a plea for a sub-specialty in dentistry, 235

X.

X-ray, value of, in diagnosis, 387

Z.

Zinc salts, test for adulterated, 455
Zsigmondy and Weiser's report on dental education, 206

